# Shaping cardiac destiny: the role of post-translational modifications on endoplasmic reticulum – mitochondria crosstalk in cardiac remodeling

**DOI:** 10.3389/fphar.2024.1423356

**Published:** 2024-10-11

**Authors:** Xiaohan Zhang, Shuqing Shi, Yihang Du, Ruoning Chai, Zezhen Guo, Chenglin Duan, Huan Wang, Yuanhui Hu, Xing Chang, Bai Du

**Affiliations:** ^1^ Department of Cardiology, Guang’Anmen Hospital, China Academy of Chinese Medical Sciences, Beijing, China; ^2^ Department of Internal Medicine, Guang’Anmen Hospital, China Academy of Chinese Medical Sciences, Beijing, China; ^3^ Faculty of Medicine, Health and Human Sciences, Macquarie University, Sydney, NSW, Australia

**Keywords:** cardiac remodeling, post-translational modifications, endoplasmic reticulum -mitochondria crosstalk, chronic heart failure, atrial fibrillation keywords

## Abstract

Cardiac remodeling is a shared pathological change in most cardiovascular diseases. Encompassing both adaptive physiological responses and decompensated pathological changes. Anatomically, atrial remodeling is primarily caused by atrial fibrillation, whereas ventricular remodeling is typically induced by myocardial infarction, hypertension, or cardiomyopathy. Mitochondria, the powerhouse of cardiomyocytes, collaborate with other organelles such as the endoplasmic reticulum to control a variety of pathophysiological processes such as calcium signaling, lipid transfer, mitochondrial dynamics, biogenesis, and mitophagy. This mechanism is proven to be essential for cardiac remodeling. Post-translational modifications can regulate intracellular signaling pathways, gene expression, and cellular stress responses in cardiac cells by modulating protein function, stability, and interactions, consequently shaping the myocardial response to injury and stress. These modifications, in particular phosphorylation, acetylation, and ubiquitination, are essential for the regulation of the complex molecular pathways that underlie cardiac remodeling. This review provides a comprehensive overview of the crosstalk between the endoplasmic reticulum and mitochondria during cardiac remodeling, focusing on the regulatory effects of various post-translational modifications on these interactions.

## 1 Introduction

Cardiac remodeling, the structural and functional transformations the heart undergoes during disease or injury, is a common pathological condition associated with various cardiovascular diseases (Omar et al., 2021). At the early stage, adaptive remodeling along with the increased cardiac output is compensated for maintaining cardiac function. Continuous stimuli, including ischemia, cardiac fibrosis, energy metabolism disorder, and the subsequent cardiomyocyte hypertrophy, are the primary maladaptive changes that contribute to cardiac disability (Metra et al., 2023; Vissing et al., 2023). Cardiac fibroblasts (CFs) undergo phenotypic changes that exacerbate the deposition of extracellular matrix (ECM), thereby enabling the differentiation of myofibroblasts that are highly proliferative and migratory (Song et al., 2024). As a result, this process orchestrates the spread of cardiac remodeling throughout the remaining cardiac chamber. Atrial remodeling refers to structural and electrophysiological abnormalities in atrial tissue that promote abnormal impulse formation or propagation, which are typically associated with atrial arrhythmias such as atrial fibrillation (AF) (Wang Y. et al., 2023). Ventricular remodeling is distinguished by ventricular wall thickening and increased mass. Systolic function may deteriorate and HF with reduced ejection fraction (HFrEF) may develop as a result of myocardial infarction and other cardiomyopathies. However, diastolic dysfunction is the initial symptom of sustained pressure overload and a major factor in heart failure with preserved heart failure (HFpEF) (González et al., 2024; Messerli et al., 2017). Ventricular and atrial remodeling can occur simultaneously or worsen one another (Pabel and Sossalla, 2022). The causal relationship between atrial and ventricular remodeling, or *vice versa*, remains unclear.

Mitochondria, the double-membrane organelles, are essential for determining energy metabolism by regulating adenosine triphosphate (ATP) synthesis through oxidative phosphorylation and the tricarboxylic acid cycle (TCA) (Polletta et al., 2015). Maintaining mitochondrial homeostasis is especially important in cardiomyocytes that consume a lot of oxygen and have high energy demands. Emerging studies indicate the critical role of extensive inter-organelles contacts in mitochondria regulation. The outer membranes of mitochondria and the endoplasmic reticulum are physically close but do not overlap, with a distance ranging from 5 to 25 nm (Csordás et al., 2006). In this spatial configuration, a myriad of proteins is localized to the mitochondria-associated membrane (MAM), preserving signal communication and functional interaction between organelles through complex molecular tethering mechanisms (Toyofuku et al., 2020). The MAM microdomain has a dynamic flux in which molecules transiently reside for milliseconds, making it susceptible to induction-driven remodeling (Obara et al., 2024). This property facilitates the maintenance of mitochondria morphology, mitophagy, organelle trafficking/positioning, mitochondria dynamics, Ca^2+^ homeostasis, and reactive oxygen species (ROS) signaling in the event of impaired cardiac energy metabolism (Sun et al., 2024).

Post-translational modifications (PTMs) are dynamic, reversible processes that respond rapidly to internal changes. Leveraging PTMs, cardiomyocytes circumvent the necessity for *de novo* protein synthesis at the transcription level, thereby conserving energy and compensating for the temporal and spatial constraints that are inherent in transcriptional regulation (Simon et al., 2021). Over six hundred types of protein modifications have been described, such as the well-known phosphorylation, ubiquitination, glycosylation, methylation, acetylation, SUMOylation, redox modifications, and neddylation. By modifying protein conformation, localization, activity, stability, and interactions with other biomolecules situated at the ER-mitochondria contact site (EMCS), the sensitivity and responsiveness of the MAM tethering system to biological processes are ultimately changed (Berger, 2007; Giacomello et al., 2020). In this review, we discuss the interaction between the endoplasmic reticulum (ER) and mitochondria during cardiac remodeling, with an emphasis on how diverse post-translational modifications influence these exchanges.

## 2 Molecular pathophysiology of ER-mitochondria crosstalk in cardiac remodeling

### 2.1 Calcium overload

**FIGURE 1:**
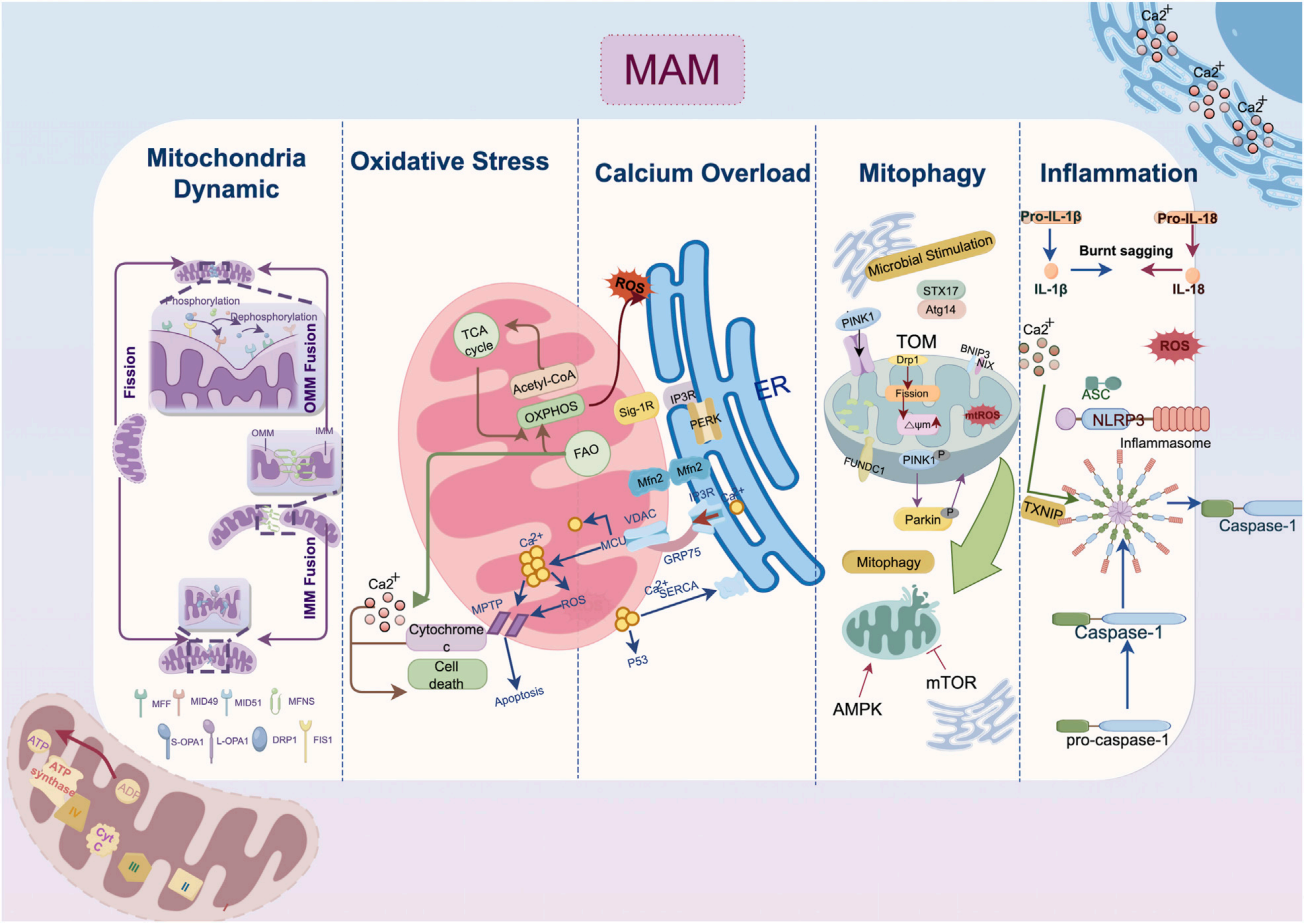
Mechanism of endoplasmic reticulum-mitochondria crosstalk in cardiac remodeling. The IP3R-GRP75-VDAC1-MCU axis regulates calcium transfer between the endoplasmic reticulum and mitochondria; MFN2 domain organization involves in outer membrane tethering; Mitophagy-related protein localized to mitochondria-associated membranes such as DRP1, FUNDC1, Pink1/Parkin jointly contribute the autophagosome formation. Abbreviation: AMPK, AMP-activated protein kinase; ASC, a caspase-recruitment domain; Atg14, autophagy-related gene 14; ATP, adenosine triphosphate; ADP, adenosine diphosphate; BNIP3, Bcl2/Adenovirus E1B 19 kDa Interacting Protein 3; Ca^2+^, Calcium ion; DRP1, dynamin-related protein 1; ER, endoplasmic reticulum; FAO, fatty acid oxidation; Fis1, fission 1 protein; FUNDC1, FUN14 domain containing 1; GRP75, glucose-regulated protein 75; IL, interleukin; IMM, inner mitochondria membrane; IP3R, inositol 1,4,5-triphosphate receptor; MAM, mitochondria-associated membrane; MCU, mitochondrial calcium uniporter; Mff, mitochondrial fission factor; Mfn, mitofusin; Mid, mitochondrial dynamics protein; mPTP: mitochondrial permeability transition pore; Nix, Nip-like protein X; NLRP3, NOD-like receptor thermal protein domain associated protein 3, NLRP3; OMM, outer mitochondria membrane; OXPHOS, oxidative phosphorylation; OPA1, optic atrophy protein 1; PERK, protein kinase RNA-like ER kinase; PINK1, PTEN-induced putative kinase 1; ROS, reactive oxygen species; SERCA2a, sarcoplasmic reticulum calcium ATPase 2a; Sig-1R, Sigma-1 receptors; STX17, syntaxin 17; TXNIP, thioredoxin-interacting protein; VDAC, voltage-dependent anion channel.

Cardiovascular remodeling is precipitated by calcium overload, which causes excitation-contraction coupling dysfunction, myocardial hypertrophy, cardiac fibrosis, and electrophysiological abnormalities. The overactivation of ryanodine receptors (RyRs), inositol 1,4,5-triphosphate receptor (IP3R) and voltage-dependent anion channels (VDACs) and declined function of sarcoplasmic reticulum calcium ATPase 2a (SERCA2a) collectively impair calcium homeostasis and cell survival (Shi et al., 2023; Dridi et al., 2020). In response to pathological stimuli, Ca^2+^ release channels such as RyRs expedite the efflux of Ca^2+^ and serve as important second messengers transmitting information among organelles (Paillard et al., 2013). As shown in Figure 1, Ca^2+^ exiting the ER traverses into mitochondria via VDAC channels on the outer membrane and the mitochondrial calcium uniporter (MCU) on the inner membrane in a mitochondria membrane potential-dependent way (Wu D. et al., 2021; Balderas et al., 2022). Excessive Ca^2+^ uptake lowers membrane potential, either directly through positive charge influx or indirectly by inducing prolonged mitochondrial permeability transition pore (mPTP) opening (Chalmers and McCarron, 2008). MAMs act as an essential platform for ER-mitochondrial Ca^2+^ transport. The classical IP3Rs-glucose-regulated protein 75 (GRP75)-VDAC complex, which is located in MAMs, is physically linked by GRP75, which directly regulates calcium transfer from the ER to the mitochondria (Wu et al., 2022). The deletion of cyclophilin D, a critical interaction with the VDAC1-IP3R1 bridge, protects cardiomyocytes from lethal hypoxia-reoxygenation injury by reducing ER-mitochondrial crosstalk and the resulting increase in mitochondrial Ca^2+^ levels (Paillard et al., 2013). Besides, Sigma-1 receptors (Sig-1Rs) form complexes with binding immunoglobulin protein (BiP) at MAMs, and when stimulated, they dissociate from BiP to bind and stabilize IP3Rs, facilitating continuous mitochondrial calcium influx (Mahamed et al., 2023; Hayashi and Su, 2007).

As a downstream response to ER stress (ERS), the scaffolding protein GRP75 is expressed, and the MAM-enriched protein levels of VDAC1, IP3Rs, and MCU are increased (Yuan et al., 2020). IP3R is expressed in three isoforms, with IP3R1 being the most prevalent in the heart and IP3R2 being the most prevalent in cardiomyocytes (Vermassen et al., 2004). Streptozotocin (STZ)-induced atrial remodeling resulted in increased IP3R1-GRP75-VDAC1 complex formation and mitochondria Ca^2+^ overload mediated by GRP75, which compromised left atrial conduction velocity (LACV), interstitial fibrosis in atrial tissue, and cardiac dysfunction (Yuan et al., 2022). These alterations collectively induce the AF occurrence. As found in the atria, the overexpression of IP3R2 in the ventricular tissue of hyperglycemia-related cardiomyopathy disturbs the Ca^2+^ flux homeostasis between the ER and mitochondria, depresses mitochondria respiration and enhances oxidative stress and cardiomyocyte apoptosis (Wu et al., 2019; Zhang N. et al., 2023; Dubois et al., 2024). On the other hand, fluctuations in the Ca^2+^ levels within the mitochondria induce the activation of matrix dehydrogenases, which subsequently promote the synthesis of ATP and oxidative phosphorylation (Denton, 2009). The mitochondrial Ca^2+^ uptake and buffering capacity may be impeded by the depletion of Ca^2+^ transfer protein, which in turn leads to energy metabolism disorder and cell death (Bravo-Sagua et al., 2019; Cárdenas et al., 2010). Given the intricate nature of ER-mitochondrial contacts, where both the over-formation and over-reduction of contact sites can be detrimental (Wu et al., 2019), delving into the upstream mechanisms that maintain the balance between ER calcium efflux and the influx to the mitochondria has become a critical target for attenuating cardiac remodeling.

### 2.2 Mitochondria dynamic dysregulation

Mitochondria are remarkably morphologically variable and subcellularly distributed, and they coordinate their many functions in cell biology to meet specific energy needs (Giacomello et al., 2020). Fission and fusion are two primary mechanisms governing mitochondria dynamics.

At the locations where the ER wraps around mitochondria, dynamin-related protein 1 (DRP1) is phosphorylated at Ser600 by calmodulin-dependent protein kinase Iα (CaMKIα), which then triggers fission by attracting DRP1 to the outer mitochondria membrane (OMM) (Duan C. et al., 2023; Taguchi et al., 2007). Then DRP1 binds to specific proteins located at the OMM, including fission 1 protein (Fis1), mitochondrial fission factor (Mff), mitochondrial dynamics protein of 49 kDa (MiD49), and mitochondrial dynamics protein of 51 kDa (MiD51) (Palmer et al., 2013). In the MAM microenvironment, the actin-nucleating proteins inverted formin 2 (INF2) maintain the DRP1 activity by driving initial mitochondrial constriction, which is subsequently followed by DRP1 polymerization (Ding et al., 2024). Mitochondria fusion begins with the docking of mitofusin 1 (MFN1) of the two OMMs and is coordinated by the interaction of optic atrophy protein 1 (OPA1) and cardiolipin triggering the inner mitochondria membrane (IMM) fusion (Giacomello et al., 2020; Ban et al., 2017). Despite MFN1 and mitofusin 2 (MFN2) sharing strikingly similar homology and structure, they present different biochemical functions. The primary function of MFN2 in fusion is to facilitate the juxtaposition of mitochondria with other organelles, particularly the ER (De Brito and Scorrano, 2008). The MFN2 splice variants, namely ER mitofusin 2 tethers (ERMIT2), facilitate inter-organelle communication by tethering the ER to mitochondria, and they work with MFN1 to regulate the transfer of Ca^2+^ and phospholipids (Naón et al., 2023; Scorrano et al., 2019).

Functional EMCS heavily rely on an intact microtubule network, disruption of which is associated with structural remodeling and contraction impairment in AF (Zhang et al., 2014; Eisner et al., 2013). Cardiac-specific MFN2 ablation reduced SR-mitochondrial tethering by 30%, resulting in lower mitochondrial Ca^2+^ uptake (Chen et al., 2012). In both tachypacing and hyperglycemia induced atrial remodeling models, EMCS decreases and the overexpression of tether protein MFN2 prevents contractile dysfunction in cardiomyocytes (Li et al., 2022). Under the pathological state of hypoxia and ischemia/reperfusion (I/R) injury, the overexpression of Opa1 and Mfn2 could inhibit fission and ROS production in ventricular cardiomyocytes thereby retarded the progression of cardiomyocyte hypertrophy and infarction (Nichtová et al., 2023; Luo et al., 2023). Similarly, MFN2-mediated enhanced tethering tends to compensate for the energetic deficit, increases cardiac resilience to injurious stress and bring about adaptive cardiac remodeling with improved contractility (Nichtová et al., 2023). Other tethering proteins in MAM conduct surveillance monitoring of quality control to preserve mitochondrial homeostasis. Lon protease 1 (LonP1), a well-conserved mitochondrial matrix protease, is essential for maintaining MAM integrity in cardiomyocytes, and its deficiency may impair cardiac function by inducing mitochondrial fragmentation via DRP1 (Li et al., 2023). However, other findings shed light on the complicated role of DRP1 regulation in MAM. For instance, syntaxin 17 (STX17), a scaffold protein that is localized on MAM, was reported to facilitate the redistribution of DRP1 on MAM, which was subsequently followed by the phosphorylation of DRP1 by S616 (Xu et al., 2023a). This cascade triggers mitochondrial fission, improving protective cardiac mitophagy (Wu et al., 2016).

### 2.3 Mitophagy suppression

Autophagy coordinates the selective self-degradation of organelles or cytoplasmic segments by encasing them in double-membrane autophagosomes, which then fuse with lysosomes for degradation, as an adaptive response to adverse conditions like nutrient deprivation and ERS (He and Klionsky, 2009). Following mitophagic stimuli, the proautophagic proteins Beclin1 (BECN1) and PTEN-induced putative kinase 1 (PINK1) are recruited at MAM, where they promote the ER-mitochondria tethering and initiate the autophagosome formation during mitophagy (Sun et al., 2024; Gelmetti et al., 2017; Quiles et al., 2023). Fission precedes mitophagy because EMCS-induced mitochondria fragmentation allows for the clearance of dysfunctional mitochondria (Kleele et al., 2021). The Ca^2+^ level of EMCS also regulates mitophagy significantly. In one respect, calcium signalling activates calcium-sensitive proteins, including LONP1, to facilitate FUN14 domain containing 1 (FUNDC1)-dependent mitophagy (Ham et al., 2023). Meanwhile, the inhibition of ER-mitochondrial Ca^2+^ transfer accompanied by the bioenergetic stress triggers the conserved sensor AMP-activated protein kinase (AMPK) located in MAM, further inactivating mechanistic target of rapamycin kinase 1 (mTOR1) and initiating mitophagy in a mTOR-independent signaling to save energy (Ahumada-Castro et al., 2019).

Under a variety of pathological stresses, including pressure overload, myocardial I/R injury, diabetes-related microvascular damage, and tachypacing-related electrical remodeling, mitophagy has been shown to be suppressed in the progression of cardiac remodeling (Li et al., 2022; Xu et al., 2023a; Chang et al., 2024a; Zhang X. et al., 2023). Notably, in transverse aortic construction (TAC) induced HF, phosphorylated-DRP1-mediated fragmentation robustly reduced in both MAM and mitochondria, which is in line with the downregulation of mitophagy biomarkers such as light chain 3II (LC3II) and Parkin (Han et al., 2017). Syntaxin 17 (STX17), a SNARE scaffold protein localized on MAMs, kickstarts the autophagy process by binding to autophagy-related gene 14 (ATG14) and facilitating autophagolysosomes formation (Arasaki et al., 2015). Overexpression of STX17 is associated with amelioration of cardiac remodeling via DRP1-dependent mitophagy (Xu et al., 2023a). As the comprehensive management of cardiac rehabilitation gradually advances, aerobic exercise is believed to ameliorate cardiac autonomic function and enhance the functional crosstalk between endoplasmic reticulum stress and mitophagy, consequently alleviating cardiac remodeling following ischemic myocardial injury (Chen et al., 2024).

### 2.4 Oxidative stress

ROS serve a vital role as secondary messengers in cellular signaling. Yet, their increased reactivity leads to oxidative stress, disrupting redox signaling and causing reversible and irreversible protein oxidation, and lipid and DNA damage (Chang et al., 2024b; Camargo et al., 2023). The association between MAMs and oxidative stress mainly involves two aspects. For one thing, Certain molecules localized to MAMs microdomain directly facilitate ROS overproduction. For example, the agonist of Sig-1R increased mitochondrial ROS significantly, potentially attributed to the binding interaction between Sig-1R and the chaperone protein Rac1 at MAM, thereby fostering the activation of NADPH oxidase (NOX) (Goguadze et al., 2019; Natsvlishvili et al., 2015). ER Oxidoreductin 1 (Ero1) plays a crucial role in maintaining ER redox homeostasis, with its two subtypes, Ero1-α and Ero1-β, predominantly localizing with 75% of Ero1-α on MAM (Gilady et al., 2010). Elevated Ero1 expression correlates with increased ROS production (Anelli et al., 2012). On the other thing, the intimate connection between organelles via MAMs leads to ROS generated in mitochondria exacerbating ERS, resulting in a vicious cycle of stress and mitochondrial dysfunction (Wang C. et al., 2023). ROS alter the activity of the SERCA as well as reduces myofilament calcium sensitivity (Van Der Pol et al., 2019). The resulting accumulation of mitochondrial Ca^2+^ leads to mitochondrial depolarization and abnormal oxidative phosphorylation, promoting the uncoupling of the mitochondrial electron transport chain from respiratory complexes I and III, further increasing mitochondrial ROS production (Goguadze et al., 2019). NOX4, enriched in MAMs of cardiomyocytes, is crucial for pro-survival signaling during stress by promoting Akt-mediated phosphorylation of inositol trisphosphate receptors, leading to inhibition of ER-mitochondrial calcium influx and mPTP-dependent necrosis (Beretta et al., 2020).

Oxidative stress, coupled with protein synthesis demands, causes the accumulation of unfolded protein to adjust and restore ER and mitochondria homeostasis. This adjustment is influenced by the redox regulation of conserved cysteine residues in the unfolded protein response (UPR) sensors protein kinase RNA-like ER kinase (PERK), inositol-requiring kinase 1a (IRE1a), and activating transcription factor (ATF) 6 (Camargo et al., 2023). In cardiac remodeling, excessive ROS production alters their deleterious regulation including activating different mediators such as transforming growth factor-β (TGF-β) and metalloproteinases (TIMP) and promoting fibroblasts transforming into myofibroblasts, enhancing ECM deposition and reducing ECM degradation (Van Der Pol et al., 2019; Wu X. et al., 2021). During the early stage of cardiac hypertrophy induced by TAC in mice, single-cell transcriptional profiling of hearts revealed a preferential accumulation of MAM-related proteins in cardiomyocytes (Luan et al., 2023). Targeting MAM-mediated oxidative stress might bring more comprehensive anti-remodeling benefits by alleviating calcium overload, inflammation, ERS and mitochondrial dysfunction.

### 2.5 Inflammation

The inflammasome contains a sensor protein and an adaptor protein, apoptosis-associated speck-like protein, which activates pro-IL-1β or pro-IL-18 (Missiroli et al., 2018). NOD-like receptor protein 3 (NLRP3) stands out as the most extensively studied and the sole inflammasome known to be linked with MAM up to now (Liu Y. et al., 2023). Multiple studies have demonstrated MAMs as a critical site for inflammasome formation. NLRP3, which is primarily expressed in macrophages, resides in the cytosol when inactive but moves to the MAM microdomain when it detects increased ROS from impaired mitochondria following a damage signal (Zhou et al., 2011). Thioredoxin-interacting protein (TXNIP), another NLRP3-binding partner redistributes to MAMs in response to oxidative stress or NLRP3 activation (Mohamed et al., 2014). During inflammasome formation, microtubules propel mitochondria towards the nucleus, leading to the subsequent apposition of a caspase-recruitment domain (ASC) on mitochondria to NLRP3 on the ER (Misawa et al., 2013). Notably, inhibition of stimulator of interferon genes (STING), an adapter protein involved in innate immunity, primarily resides in the ER and MAM has been found to decrease NLRP3 activation (Liu et al., 2024). Moreover, as a crucial physical function of MAM microdomain, lipid metabolism is also an important contributor to inflammation response. Thus, caveolin-1(CAV1) modulating cell metabolism that is highly concentrated in the cardiomyocyte MAM while preserving its integrity, exhibits anti-inflammatory characteristics (Liu et al., 2024; Liu I.-F. et al., 2023). Conclusively, the MAM-mediated regulation of Ca^2+^ and lipid homeostasis between the ER and mitochondria is pivotal for the sustained activation of NLRP3 inflammasomes. This inflammation response is linked to cardiac morphological and electrophysiological remodeling, mostly instigated by the aberrant Ca^2+^ release from the SR (Ajoolabady et al., 2022).

## 3 Post-translational modifications and ER-mitochondria-related cardiac remodeling

### 3.1 Phosphorylation

Phosphorylation is the process by which kinases add or remove γ-phosphate groups (PO_3_^2-^) to/from amino acid residues of substrate proteins, primarily occurring on Serine (Ser), Threonine (Thr), and Tyrosine (Tyr) residues (Battaglioni et al., 2022). Approximately 30% of proteins undergo phosphorylation with hundreds of kinases collectively participating in various signaling pathways that modulate the functional crosstalk and structural integrity between the ER and mitochondria, consequently targeting cardiac remodeling (Xu et al., 2020).

**FIGURE 2:**
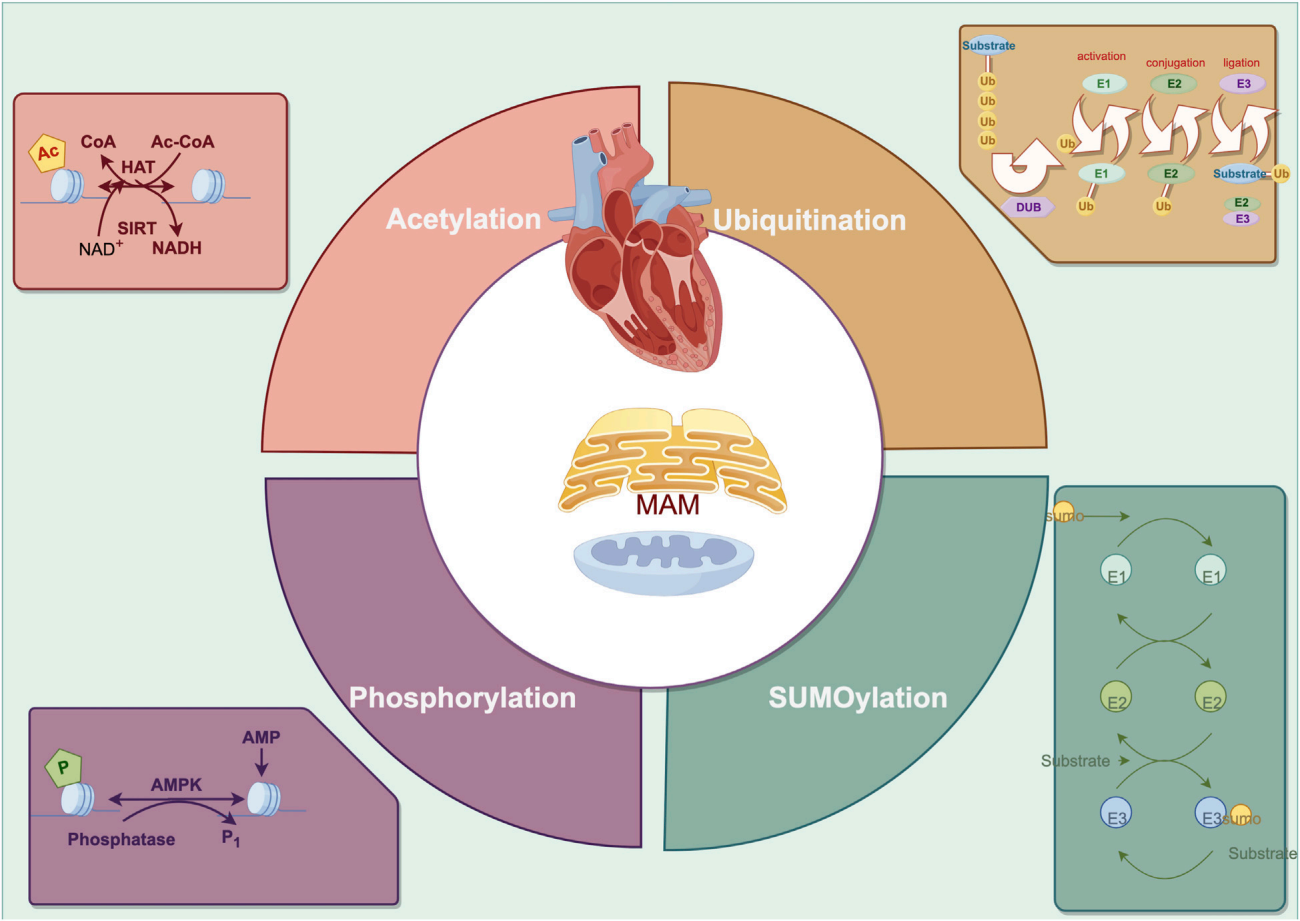
The primary posttranslational modification in cardiac remodeling regarding endoplasmic reticulum-mitochondria crosstalk.

As shown in Figure 2 and Table 1, adenosine 5′-monophosphate (AMP)-activated protein kinase (AMPK) is a key kinase in regulating cellular energy metabolism. Its chronic activation prevents cardiac remodeling by restoring mitochondrial and cardiac ultrastructure (Shimizu et al., 2018). The AMPK phosphorylation at Thr172 significantly decreased in hyperglycemia-related hearts and concomitantly associated with an increase of FUNDC1, which induces aberrant MAM formation reversibly (Wu et al., 2019). Extracellular signal-regulated kinases (ERKs) and c-Jun N-terminal kinase (JNK) are conserved kinase subfamilies of the mitogen-activated protein kinases (MAPK) family primarily regulates stress signaling. Hyperactivation of ERK1 phosphorylation was observed downstream of MFN2 ablation, which fails to tether the two organelles, exacerbating pathological cardiac hypertrophy (De Brito and Scorrano, 2009). The protein kinase C (PKC) acts as a direct inducer of the cardiomyocyte growth response by regulating phosphorylating inhibitor-1 (PP-1), correspondingly, the decrease PP-1 activity leads to enhanced SERCA2 activity and cardiac contractility in PKC knockout mice (Newton et al., 2016). As the most classical target in MAM regulating calcium overload, SERCA2 exerts its protective mechanism more than promoting activation. Inhibition of glycogen synthase kinase-3β (GSK3β) phosphorylation at the Ser663 site of SERCA2 reduces Ca^2+^ dyshomeostasis, cell death and infarct size after ischemia-reperfusion injury (Gonnot et al., 2023; Gomez et al., 2016). Under chronic overload induced by TAC, cyclin-dependent kinase-1 (CDK1) is recruited onto MAM to phosphorylate DRP1 at Ser616 and promote DRP1-mediated mitophagy (Xu et al., 2023a). Similarly, the activation of cAMP- dependent protein kinase (PKA) presented pro-survival effects during the early stage of ERS, which mediates the DRP1 phosphorylation at Ser637 fulfilling a dual objective to reinforce protein folding at ER and enhance ER-mitochondria Ca^2+^ transfer (Bravo-Sagua et al., 2019; Lynes et al., 2013). Oppositely, silencing PKA decreases Mfn2 Ser442 phosphorylation while upregulating the expression of Mfn2, leading to the restoration of MAM tethering and mitochondrial fusion and inhibition of cell apoptosis in failing right ventricular (Dasgupta et al., 2021). Yet, the controversially bidirectional regulatory network modified by phosphorylation in cardiac remodeling requires more elaborated investigation.

**TABLE 1:** The Posttranslational modification of regulating the ER-mitochondrial crosstalk in cardiac remodeling.

PTM	Protein substrate	Modifying enzymes	Biological process	Stimuli
Phosphorylation	FUNDC1	AMPK	Ca^2+^homeostasis	STZ-Hyperglycemia
Phosphorylation	MFN2	ERK1	MAM structure	-
Phosphorylation	SERCA2	GSK3β	Ca^2+^homeostasis	Ischemia-reperfusion
Phosphorylation	DRP1	CDK1	Mitochondrial autophagy	TAC
Ubiquitination	CHOP	Parkin	ERS	TAC
Ubiquitination	IP3R2	Rnf170	Ca^2+^homeostasis	STZ-Hyperglycemia
SUMOylation	Drp1	SENP3	ERS/apoptosis	Ischemia-reperfusion
SUMOylation	FIS1	SENP1	Mitochondrial homeostasis	Hypoxia
SUMOylation	NDP52	MUL1	Mitochondrial autophagy	Radiation
SUMOylation	SERCA2a	SUMO1 and Ubc9	Ca^2+^homeostasis	TAC
SUMOylation	—	UBC9	Autophagy	TAC
Acetylation	SERCA2a	p300/SIRT1	Ca^2+^homeostasis	Pressure overload and ischemia
Acetylation	DRP1	SIRT 3	Mitochondrial fission	High-fat diet
Acetylation	Pink1/Parkin	SIRT 3	Mitochondrial autophagy	Hypertension
Acetylation	ATAD3A	SIRT 3	Ca^2+^homeostasis	Isoproterenol stimulation

ATAD3A, AAA-domain containing protein 3A AMPK, 5′-AMP-activated protein kinase; CDK1, cyclin-dependent kinase-1; CHOP, C/EBP, homologous protein; DRP1, dynamin-related protein 1; ERK, Extracellular signal-regulated kinases; FIS1, fission 1 protein; FUNDC1, FUN14 domain containing 1; GSK3β, glycogen synthase kinase-3β; IP3R, inositol 1,4,5-triphosphate receptor; MULAN, mitochondrial-anchored protein ligase; PTM, Posttranslational modification; SENP, Sentrin-specific proteases; SERCA2a, sarcoplasmic reticulum calcium ATPase, 2a; SIRT, sirtuins; SUMO, small ubiquitin-like modifier; UBC9, ubiquitin-conjugating enzyme 9.

### 3.2 Ubiquitination

The ubiquitin-proteasome system (UPS) largely facilitates the selective elimination of particular aberrant protein molecules within the cell. Ubiquitin E3 ligases serve as the rate-limiting enzymes of the UPS conferring substrate specificity, whereas deubiquitinating enzymes (DUBs) regulate the dynamic and reversible equilibrium of ubiquitination (Kitamura, 2023). Impaired UPS activity is commonly found in various cardiac remodeling processes, with the MAM playing a crucial role in UPS degradation (Hu et al., 2018).

Ubiquitination disrupts the reticulon homology domain of autophagy receptors in the ER, which permits the formation of dense receptor clusters via interdomain contacts; these clusters subsequently bind to LC3II, allowing endoplasmic reticulum autophagy to meet energy metabolic demands (González et al., 2023). Ubiquitination often regulates protein quality by interacting with phosphorylation. When mitochondria are damaged and lose their polarization, PINK1 accumulates on the OMM and undergoes autophosphorylation at the Ser228, recruiting and phosphorylating the E3 ubiquitin ligase Parkin (Eldeeb et al., 2024). In the MAM, PINK1 acts as an upstream regulator of autophagy genes, balancing mitophagy and ER-phage through its interaction with various E3 ligases in the ubiquitin degradation pathway (Wang R. et al., 2023).

The essential protective mechanism for regulating the connection between the ER and mitochondria at the posttranslational level is to maintain the equilibrium of tethering and calcium signal transduction in the cell. E3 ligases located in MAM, such as Parkin and membrane-associated RING-CH5 (MARCH5), mitochondrial-anchored protein ligase (MULAN) mediate the phosphorylation-dependent ubiquitination and degradation of other component proteins, such as Mfn2, MCU, and VDAC1, which regulates the organelles tethering and alleviates mitochondria calcium overload and dysfunction (Toyofuku et al., 2020; Wu et al., 2022; Luo et al., 2023; Xu et al., 2023b; Zungu et al., 2011; Ambivero et al., 2014). In TAC-induced cardiac remodeling, C/EBP homologous protein (CHOP) is a substrate for Parkin ubiquitination, and its degradation by Parkin can alleviate the ERS that triggers cell apoptosis (Han et al., 2017). Conversely, in Parkin knockdown mutants, damaged mitochondria can activate the PERK-induced unfolded protein response and ERS signaling (Popovic et al., 2021). On top of that, in STZ-induced cardiac remodeling, the increase of FUNDC1 mediated by hyperglycemia interacts with IP3R2 and inhibits IP3R2 ubiquitination and proteasomal degradation (Wu et al., 2019).

### 3.3 SUMOylation

The SUMO system, a transitory process that covalently attaches to lysine residues in target proteins, plays a crucial role in determining the physiologic changes in cardiomyocytes (Hotz et al., 2021). Sentrin-specific proteases (SENPs) are well-studied deSUMOylating enzymes involved in regulating mitochondria dynamics, mitophagy and calcium overload (Prudent and McBride, 2017; Xiao et al., 2022). Markedly, the process of mitochondria fission and the reduction of SENP1 are observed, resulting in an obvious dissociation of mitochondria from the ER and worsening of cardiac remodeling (Zungu et al., 2011; Figueroa-Romero et al., 2009; Buccarello et al., 2020). However, the protective effect of SUMO1-mediated SUMOylation which stabilized LV volumes and maintained cardiac function has also been reported (Tilemann et al., 2013). For the high dynamics and duality, investigating the additional mechanism of SUMO modification is of vital importance.

In I/R animal models, the SENP3 upregulation impaired the crosstalk between mitochondria and ER in a DRP1-dependent manner suggested by the ERS and mitochondria-mediated apoptosis, while the SENP3 knockout rescued the pathological remodeling (Anderson and Blackstone, 2013; Gao et al., 2018). Interestingly, the interaction of DRP1 and DRP1-FIS1 has been demonstrated crucial role in myocytes and fibroblasts with FIS1 acting as the driving force of mitochondria fission (Yu et al., 2019). Under hypoxia-induces right ventricular remodeling, translocation of SENP1 to mitochondria but not the change of expression level deSUMOylates FIS1 to assemble with MFN2 and mitochondrial gatekeeper VDAC1, making it preserve mitochondrial integrity, MAM formation, and cellular calcium homeostasis (Zhou et al., 2023). In radiation-induced cardiac hypertrophy, low-dose X-rays trigger SUMOylation, inhibiting PINK1 recruitment to MAM and consequently impeding the mitophagy through SUMO2 (Gao et al., 2024). Endogenous SUMO1 expression is decreased in experimental animal models and human CHF (Chang and Yeh, 2020). In the SUMO1 transgenic CHF pig model, similar improvements in cardiac function were observed as in the SERCA2a transgenic CHF model (Kho et al., 2011). Further investigation revealed that SUMO1 regulates SERCA2a at the K480R and K585R sites, enhancing its enzymatic activity as well as stability, enhancing positive inotropic effects on myocardium (Lai et al., 2023). Additionally, SUMO E2 ligase ubiquitin-conjugating enzyme 9 (UBC9) levels elevated in response to TAC and other cardiomyopathic stress, which specifically enhance the removal of misfolded proteins during the ERS and sustain mitochondria protein quality control by increasing autophagic flux (Gupta et al., 2014; Gupta et al., 2016).

### 3.4 Acetylation

Post-translational acetylation catalyzes the site-specific N-ε-acetylation of lysine residues’ ε-amino group, and lysine acetyltransferases (KATs) and lysine deacetylases (KDACs) are responsible for reversible changes (Menzies et al., 2016). Specifically, class III KDACs are composed of nicotinamide adenine dinucleotide^+^ (NAD^+^)-dependent sirtuins (SIRTs), with SIRT3 being the primary deacetylase in the mitochondria quality control system, which impedes atrial and ventricular remodeling (Karamanlidis et al., 2013; Weinert et al., 2015; Liu et al., 2022). The advancement of myocardial energetics, redox balance and calcium homeostasis are the hallmarks of targeting SIRTs-dependent acetylation in failing heart (Lee and Tian, 2015).

Pressure overload and ischemia are associated with the elevation SERCA2a acetylation at Lysine (K492) and K514 mediated by histone acetyltransferase (p300), resulting in the SERCA2a activity suppression, while the interaction of SIRT1 and SERCA2a can restore SERCA2a activity through deacetylation (Gorski et al., 2019; Gorski et al., 2023). High-fat diet causes myocardial damage and DRP1-mediated fission by promoting DRP1 acetylation at K642, which is coupled with Drp1 translocation to MAMs and binding VDAC1 (Hu et al., 2020). SIRT3 enhances the oligomerization level of AAA-domain containing protein 3A (ATAD3A) by binding and deacetylating ATPase family on K134, an essential regulator for MAM formation localized to MAMs in heart tissue (Frazier et al., 2021), thus decreasing the excessive MAMs formation induced by isoproterenol and the subsequent calcium overload mediated by IP3R1-GRP75-VDAC1 complex (Frazier et al., 2021; Li et al., 2024). Besides, PINK1/Parkin was demonstrated as the potential substrate of SIRT3, for their acetylation increased in response to hypertension-related cardiac remodeling and SIRT3 overexpression restore the mitophagy and angiogenesis (Wei et al., 2017).

### 3.5 Pharmacological intervention

By regulating protein function, stability, and signaling, the pharmacological application of PTMs in cardiac remodeling gives new avenues for cardiac metabolism, stress response, fibrosis, and apoptosis. Research indicates various natural products may modify post-translational modifications, thereby affecting ER-mitochondria signaling and reversing cardiac remodeling. Hesperadin was identified as a CaMKII inhibitor through a small-molecule kinase inhibitor library and high-throughput screening, resulting in a significant reduction in CaMKII phosphorylation levels, thereby mitigating DNA damage in cardiomyocytes induced by CaMKII-δ (Zhang et al., 2022). Additionally, Ginsenoside Rd was shown to be an effective CaMKII inhibitor and to mitigate MI injury by promoting mitochondrial biogenesis and alleviating oxidative stress and calcium accumulation (Cui et al., 2023). Ginsenoside Rg3 also counteracted the impact of CHF on SUMO1 and SENP1, facilitating the translocation of SUMO1 from the nucleus to the ER and its co-localization with SERCA2a within the ER (Liu et al., 2021). The HMG-CoA reductase degradation protein 1 (HRD1) is an ER-transmembrane E3 ubiquitin ligase (Doroudgar et al., 2015). The antiepileptic medication zonisamide enhanced the degradation of ER-associated proteins (ERAD) by upregulating Hrd1 expression and suppressing ER stress, therefore preventing cardiac hypertrophy (Wu Q. et al., 2021).

However, the challenge of converting these molecular insights into therapies is inevitable. Firstly, designing drugs that precisely target specific PTMs without affecting related pathways is challenging due to the complexity of PTMs networks and their involvement in various cellular functions. Secondly, the dynamic and reversible nature of PTMs complicate the attainment of sustained therapeutic effects, necessitating continuous modulation. Thirdly, efficiently delivering PTM-modulating agents to specific tissues or organs, such as the heart, without affecting other areas, poses technical and pharmacokinetic challenges.

## 4 Future perspectives

Potential future research could concentrate on the development of pharmacological agents that specifically target key proteins involved in ER-mitochondria interactions, such as the VDAC-IP3R-GRP75 complex and MFN2, to alleviate calcium overload, oxidative stress, and inflammation, thereby preventing or attenuating cardiac remodeling.

Investigating the therapeutic potential of modulating PTMs could lead to the development of novel treatment strategies for cardiovascular diseases. While several critical PTM-regulating enzymes and pathways have been identified, future research should persist in employing techniques like high-throughput library screening to discover new PTM targets associated with cardiac remodeling. Functional validation by genetic and pharmacological methods is crucial for discovered PTMs, particularly those associated with ER-mitochondria crosstalk. Integrative multiple omics methodologies, such as proteomics and modified proteomics, allowing for mass spectrometry to qualitatively and quantitatively analyze modified proteins or peptides, facilitates the more accurate identification of crucial regulatory mechanisms and elucidates the significance of alterations in protein expression and modification levels within the organism (Duan S. et al., 2023). Through the combination of multi-dimension, these methods collectively clarify biological response mechanisms. Advanced computational modeling and bioinformatics tools will help map PTM crosstalk networks in cardiac remodeling and identify novel therapeutic targets. Moreover, certain modifier proteins present on the cell surface are prone to secretion into biofluids like blood or urine, making them accessible for non-invasive testing (Wozniak et al., 2020). This would allow for early-stage detection and monitoring of treatment response as clinical translation.

## 5 Conclusion

Cardiac remodeling encompasses both pathogenic and adaptive changes induced by sustained stimulation. The classification can be further divided into dilated remodeling and hypertrophic remodeling (Martínez-Legazpi et al., 2014). Hypertrophic remodeling often occurs in conditions such as hypertension, AF, and metabolic disorders, whereas dilated remodeling involves increased cardiac volume and thinning of the cardiac wall, commonly associated with ischemic heart disease (Wang Y. et al., 2023; Chang et al., 2024a; Ritterhoff et al., 2020; Chang et al., 2023). The interaction between the ER and mitochondria constitutes the predominant contact site between organelles in mammalian cells, revealing important regulatory mechanisms represented by organelle tethering and calcium signaling during cardiac remodeling (Nichtová et al., 2023; Heinrich et al., 2021). This review outlines SERCA2, VDAC1, IP3R, and other calcium signaling proteins as the most extensively studied targets in the ER-mitochondria functional interaction. As substrates undergo modifications, such as phosphorylation, ubiquitination, and SUMO, which greatly affect the process of cardiac remodeling. This is expected to establish a theoretical foundation for understanding PTMs in the crosstalk between the ER and mitochondria during cardiac remodeling.
